# The relationship between the PICC tip position and weight gain, length growth of premature infants under ultrasonography: a correlation analysis study

**DOI:** 10.3389/fmed.2023.1200033

**Published:** 2023-06-14

**Authors:** Xiaojun Tao, Xianhong Zhang, Jianhui Wang, Yanhan Chen, Xuexiu Liu

**Affiliations:** ^1^Department of Neonatology, Children's Hospital of Chongqing Medical University, Ministry of Education Key Laboratory of Child Development and Disorders, National Clinical Research Center for Child Health and Disorders, China International Science and Technology Cooperation Base of Child Development and Critical Disorders, Chongqing Key Laboratory of Pediatrics, Chongqing, China; ^2^College of Nursing, Chongqing Medical University, Chongqing, China

**Keywords:** ultrasonography, location, premature infant, peripherally inserted central catheter, weight, length, position

## Abstract

**Objective:**

This study aimed to analyze the correlation between PICC tip position and weight/length changes in preterm infants in different positions using ultrasonography.

**Methods:**

The study is a prospective before and after self-control clinical trial. The study analyzed the distance between the PICC tip and the entrance of the heart under ultrasonography for premature infants who underwent PICC insertion. The infants were positioned and tracked weekly, and their weight and length were recorded. The Spearman rank correlation test was used to analyze the relationship between the displacement distance of the PICC tip under ultrasonography in different positions and weight/length changes.

**Results:**

A total of 202 premature infants were included in the study, and 100% of them experienced changes in the PICC tip position. During the first week, 134 (66.33%) cases in a flexed position and 153 (75.74%) cases in a straight position showed displacement of the catheter toward the heart. The displacement distance of the tip during catheter retention was significantly correlated with weight change (*r*_s_ = 0.681/0.661, *P* < 0.05) and length change (*r*_s_ = 0.629/0.617, P < 0.05). In the third and fifth weeks, weight increased by 451 ± 178 and 750 (715–975) g, length increased by 1.50 (1.00–2.12) and 3.00 (2.00–3.70) cm, the catheter moved 1.27 ± 0.89 and 2.23 ± 0.95 cm, respectively, in a flexed position.

**Conclusion:**

The PICC tip position in preterm infants is influenced by weight and length changes. It is crucial to use ultrasonography to track and locate the catheter within the first week of placement and to increase the frequency of catheter localization starting from the third and fifth weeks. The flexed position is recommended during catheter localization.

## Introduction

A peripherally inserted central catheter (PICC) is a technique for inserting a catheter through peripheral veins so that the catheter tip is placed in the superior vena cava (SVC) or inferior vena cava (IVC) to establish a safe and stable infusion pathway. The PICC has the characteristics of safety, long retention time, and low associated infection rate, providing ideal venous access for newborns, especially for premature infants with a severe nutritional deficiency (1). During the indwelling catheter, ensuring the catheter tip within the vena cava is critical because malposition may induce adverse outcomes such as infectious endocarditis, atrial fibrillation, and pleural effusion (2–4). Therefore, it is necessary to regularly track and locate the PICC tip in premature infants from the first location to prevent displacement (5).

Currently, PICC positioning mainly includes body surface measurement, intracavitary ECG, chest radiography (CR), and ultrasonography (US). The body surface measurement method and intracavitary ECG positioning are only used for the first catheter placement, and cannot continuously track the catheter position (6, 7), while CR has been considered the “gold standard” for confirming the sites of catheter tips, it has several drawbacks such as non-dynamic and retrospective imaging, ionizing radiation, and longer time consumption (8). The traditional operation process did not locate and track the PICC during the indwelling catheter. In the case of suspected complications, the CR would be used for location judgment. However, due to the problem of the clinical use of CR, the implementation of PICC location and tracking was hindered. At this time, the advantages of PICC tip location and tracking under US were also highlighted (9–11). Research shows that US can not only clearly display the position of the PICC tip in the vena cava (12) but it also can timely detect and guide the correction of catheter ectopic (13, 14). US can directly display the superior and inferior vena cava and its right atrium entrance, which provides a basis for accurately determining the position of the catheter tip in the vena cava (15).

The catheter displacement of premature infants is caused by body length and weight growth. Weight and length are the most intuitive indicators to measure growth, and the measurement is simple and accurate (16, 17). Studying the correlation between the change of catheter tip position in different body positions will help to provide the theoretical basis for the tracking of PICC catheter position. However, only a few studies have reported on the application of US in the neonatal intensive care unit (NICU).

Hence, in this study, we enrolled a consecutive series of 210 premature infants, aiming to understand more about the values of US in tracking and locating the position of catheter tip under different body positions.

## Methods

### Estimation of sample size

We used the “Confidence Intervals for Kappa” in PASS15 software to estimate the sample size of this study. According to clinical experience and pre-experimental results, the kappa coefficient is about 0.806, and its standard deviation is 0.12. If the class I error of the relevant parameters is set as 0.05 (α = 0.05) with an allowable error of 0.05 (δ = 0.05), the calculated sample size is 89 children. With the addition of 10% sample loss, at least 98 patients are required.

### Participants

Premature infants who were hospitalized in the Department of Neonatology, Children's Hospital Affiliated to Chongqing Medical University from September 2022 to March 2023, and needed PICC catheterization were selected as the study subjects. The inclusion criteria were as follows: premature infants requiring PICC catheterization; the physical condition of the infant able to withstand US examination; the bleeding and clotting time being normal; no serious contraction or collapse of peripheral blood vessels; PICC retention time >1 week; and parents are informed and signed informed consent. The exclusion criteria were as follows: congenital heart disease or other cardiovascular diseases; immunodeficiency disease; severe intestinal inflation, unable to determine the position of catheter tip; the local skin of the ultrasonic observation window being incomplete, with skin lesions or infection; and transfer, abandonment of treatment, or death. This study was approved by the hospital ethics committee (2022-369); Clinical trial registration number (ChiCTR220064003).

### Placement of PICC

PICC placement was performed by two nurses with PICC operation qualification. Briefly, the neonate was placed in an incubator. Catheterization was performed with a puncturing kit containing 26 GA (1.9 F) single-lumen PICC catheters according to the neonatal PICC catheterization operation specifications.

According to the specifications, it should be avoided placing the catheter tip in the heart of neonates and infants (15). The optimal tip position complied with the recommendation of the 2016 guidelines by the American Infusion Nurses Society (INS), i.e., the safest PICC tip should be located within the lower third of SVC or just below the IVC-and-right-atrial junction (3, 18, 19).

### Locating catheter tips by US

**Figure 1:**
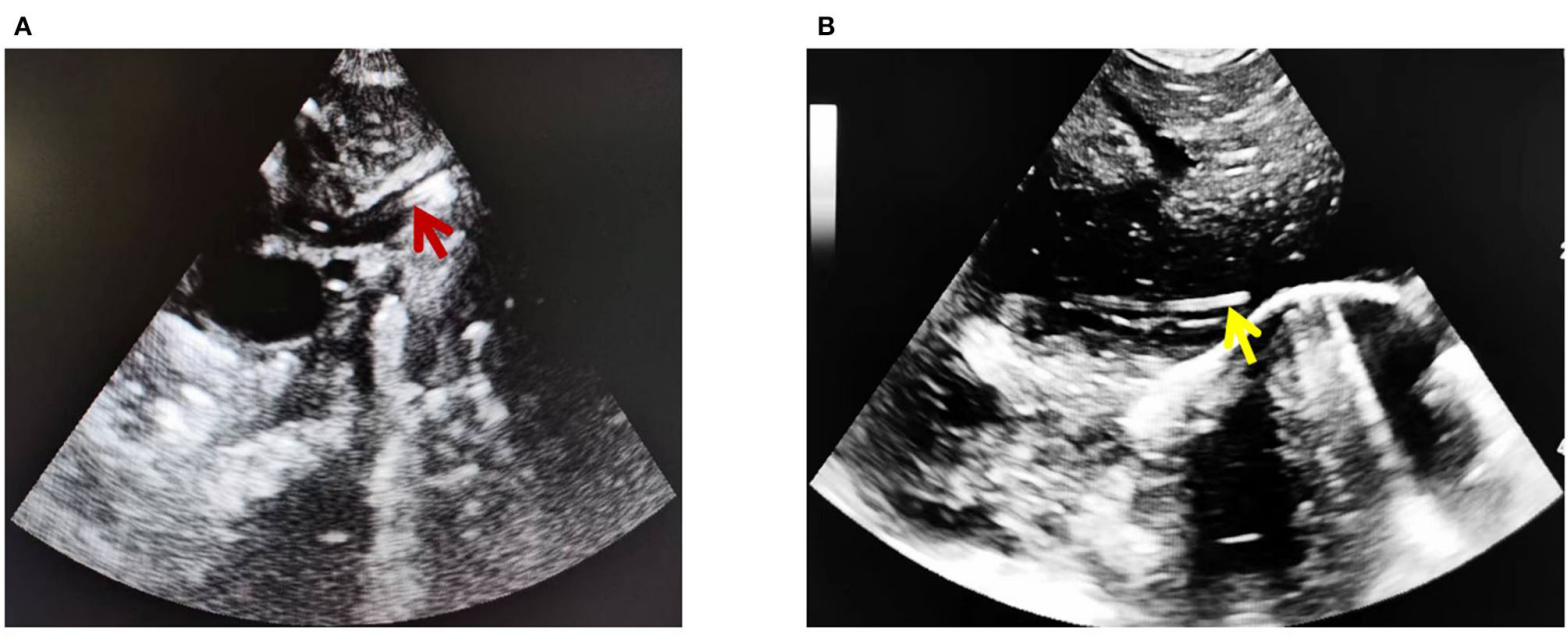
PICC ultrasonic imaging of vena cava. **(A)** Superior vena cava (red arrow). **(B)** Inferior vena cava (yellow arrow).

US is conducted under a LOGIQ e color Doppler ultrasonic diagnostic system (6S and 8C probes, GE company, USA) by two research members who have at least 5 years of experience in ultrasonic PICC positioning. The ultrasonic probe was set at the midline of the subxiphoid region or at the parasternal line of the right subclavicle region. A hyperechoic “equal sign'-like or sandwich-like structure would be detected within the vena cava, which represents the inserted line. In detail, for clearly viewing the “equal sign” like echoes of the catheter tip in SVC, the probe was placed longitudinally at the 2nd−3rd intercostal spaces on the right of the sternum to delineate the long axis of the aortic arch and the short axis of SVC and then rotated clockwise for about 15° and tilted slightly to the right to show the long axis of SVC and the right atrial entrances of SVC and IVC (Figure 1A). Subsequently, the distance between the tip and the right atrial inlet was measured, and improper tip position was US-guided readjusted. For clearly viewing the “equal sign”-like echoes of the catheter tip in IVC, the probe should be placed longitudinally at the midsagittal position of the subxiphoid region and scanned along the inferior rib to delineate the IVC and right atrial inlet (Figure 1B). The tip-to-atrium distance was measured, and improper tip position was readjusted.

### Observation and analysis

The puncture site, the distance between the catheter tip and the right atrium inlet, was measured under US on the day of catheterization and every other week after catheterization. Recording the weight and length at the time of the PICC catheter was done every week, and the increase in weight and length after inserting the PICC catheter was calculated. To calculate the correlation between the increase in weight and length of premature infants and the displacement distance of the PICC tip, all data were entered by two PICC specialist nurses, and the results with differences were reviewed and agreed upon by two people. Two people will input all data into the computer, and all electronic data will be checked by a third person.

### Statistics

In this study, SPSS 24.0 software was used to statistically process the data. The counting data is expressed in cases and percentage (%). The measurement data conforming to normal distribution are described by mean ± standard deviation, and the measurement data of non-normal distribution are expressed by median and quartile spacing; The correlation between weight gain and length growth and PICC tip shift was analyzed by the Spearman rank correlation analysis, and a *P*-value of < 0.05 was considered to be a statistically significant difference.

## Results

### Patients

**Figure 2:**
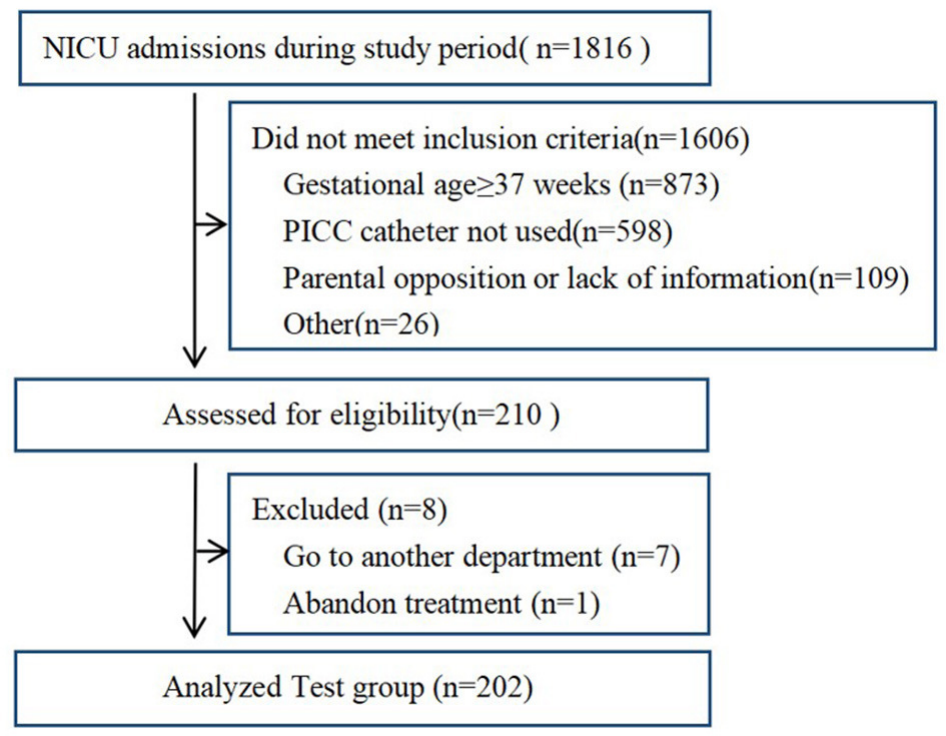
Flowchart of the study population.

A total of 1,816 newborn infants were screened between September 2022 to March 2023, of which, 1,606 did not meet inclusion criteria, including 873 of gestational age (GA) ≥ 37 weeks; thus, 598 PICC catheters were not used, 109 parents of preterm infants declined to participate, and 26 are excluded for other reasons. Finally, 210 premature infants enrolled, of which eight of them dropped out (seven were transferred to another department and one abandoned treatment). Finally, 202 finished the study before and after self-control trials and were included in the final analysis (Figure 2). The number of involved preterm infants reached the calculated sample size.

Of the included neonate, 121 were boys and 81 were girls. A total of 69 cases received invasive mechanical ventilation, 101 received non-invasive mechanical ventilation, and 32 were not on a ventilator. Upper limb catheterization was performed in 11 cases, including one case of the superficial temporal vein, eight cases of the basilic vein, and two cases of the cephalic vein. Lower limb catheterization was performed in 191 cases, including 128 cases of great saphenous vein and 63 cases of superficial femoral vein. The mean gestational age was (31.75 ± 2.83) weeks, the age at the time of catheterization was (3.09 ± 7.46) days, and the weight on the day of catheterization was (1,758.54 ± 591.58) g.

### Catheter indwelling time, weight gain, length growth, and PICC tip displacement distance in flexion and extension positions during catheter indwelling

**Figure 3:**
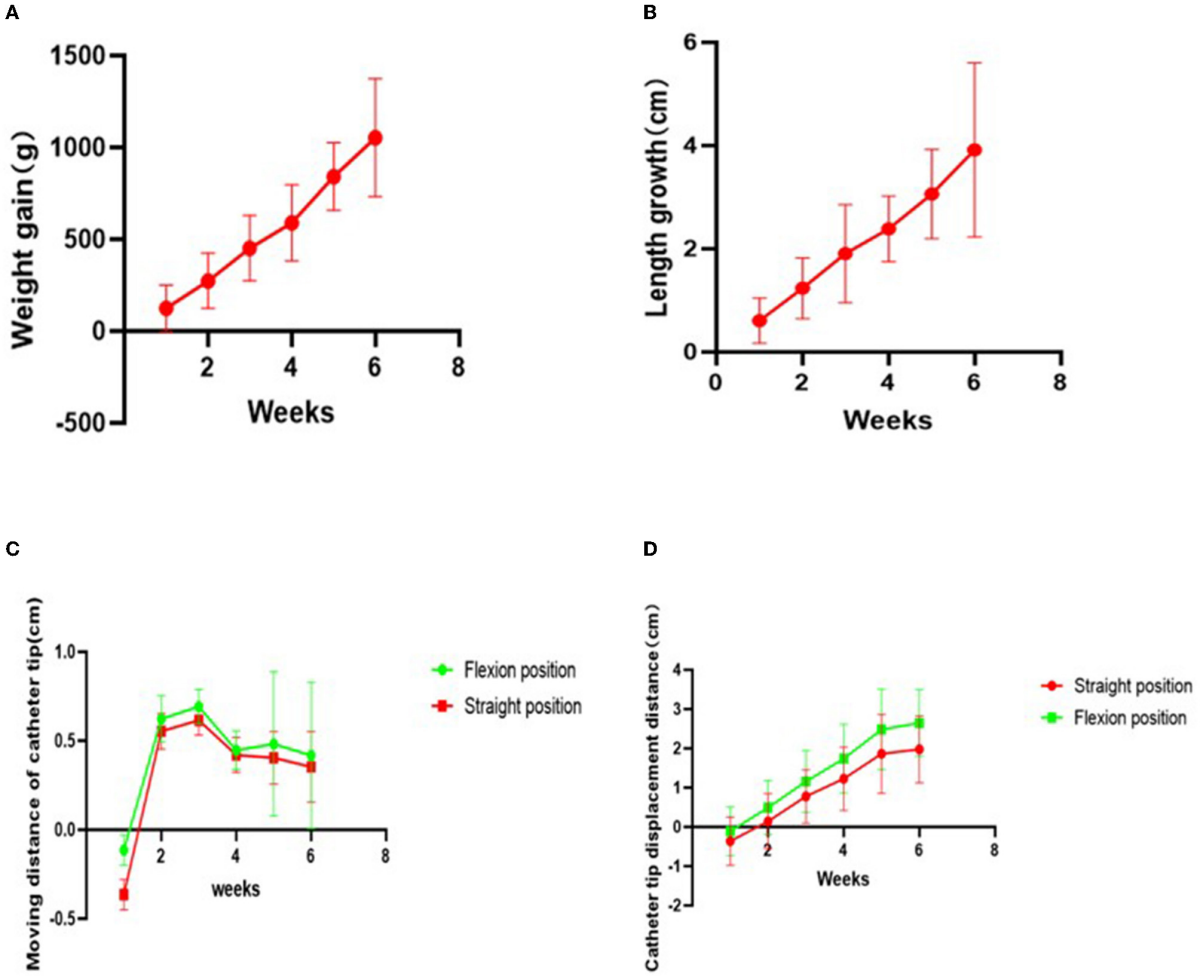
**(A)** Weight gain; **(B)** length growth; **(C)** tip displacement distance of weekly in flexion position and straight position; **(D)** Tip displacement distance in flexion position and straight position.

The results showed that the PICC catheterization time was 15 days, with a weight gain of 230 g and a length growth of 1 cm. The catheter tip displacement was 0.33 and 0.09 cm, as shown in Table 1. From the time of catheterization to the 3rd week, the weight gain was 451 ± 178 g, the length growth was 1.50 (1.00–2.12) cm, and the catheter displacement was 1.27 ± 0.89 cm. By the fifth week, the weight gain was 750 (715–975) g, the length growth was 3.00 (2.00–3.7) cm, and the catheter displacement was 2.23 ± 0.95 cm. The weight and length growth during the catheterization period is shown in Figures 3A, B, respectively. The tip displacement distance in each week in the flexion position was −0.06 ± 0.79, 0.57 ± 0.41, 0.76 ± 0.20, 0.36 ± 0.23, 0.58 ± 0.52, and 0.32 (0.17–0.57) cm; in the straight position, it was −0.31 ± 0.77, 0.51 ± 0.30, 0.62 ± 0.20, 0.36 ± 0.29, 0.43 ± 0.17, and 0.35 ± 0.18 cm, as shown in Figure 3C. The tip displacement in the flexion and straight positions from the beginning of catheterization was −0.06 ± 0.79, 0.51 ± 0.88, 1.27 ± 0.89, 1.64 ± 0.82, 2.23 ± 0.95, 2.64 ± 0.84 cm and 0.31 ± 0.77, 0.20 ± 0.91, 0.82 ± 0.80, 1.18 ± 0.87, −1.62 ± 0.93, 1.98 ± 0.85 cm, respectively, as shown in Figure 3D. In the first week, 134 (66.33%) of the flexion position and 153 (75.74%) of the straight position were displaced to the heart direction, 0.37 (0.26–0.56)/0.66 (0.45–0.87) cm respectively. The fluctuation of the data of catheter tip displacement distance measured in different positions showed that the tip displacement distance was 0.23 (0–0.97) cm in flexion position and 0.25 (0–0.98) cm in the straight position from the second week. The small fluctuation range in the flexion position indicates that the stability of the flexion position is higher. There were five patients with edema. After the edema subsided, the catheter tip was tracked and found to be displaced 1.04 ± 0.24/1.43 ± 0.17 cm in the flexion and straight position of the catheter tip to the heart direction in the first week.

**Table 1:** Conditions of catheter retention period.

**Item**	**Min and max**	**Median and interquartile spacing**
Catheter retention time (days)	8–49	15 (11–20.25)
Weight gain (g)	−250 to 1,660	230 (100–370)
length growth (cm)	0–7	1 (1–2)
PICC tip displacement in flexion position (cm)	−1.28 to 4.05	0.33 (−0.2 to 1.03)
PICC tip displacement in straight position (cm)	−2.0 to 3.41	0.09 (−0.41 to 0.70)

### Correlation between weight gain and length growth and PICC catheter tip displacement in flexion and straight positions

**Figure 4:**
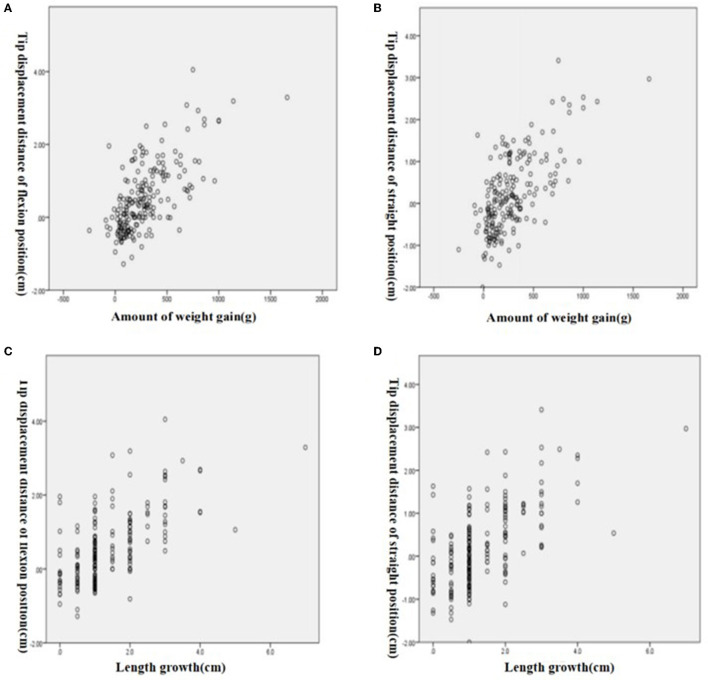
Correlation between weight gain and tip displacement distance. **(A)** Flexion position. **(B)** Straight position. Correlation between length grow and tip displacement distance. **(C)** Flexion position. **(D)** Straight position.

Using the Spearman rank correlation analysis, different positions had different correlations between the distance of catheter tip displacement and weight gain. The correlation coefficient in the flexion position was *r*_s_= 0.681, with a *P*-value of < 0.05, and *r*_s_= 0.661, with a *P*-value of < 0.05 in the straight position, indicating a significant correlation between the two positions as shown in Figures 4A, B. The correlation between the distance of catheter tip displacement and length growth was also different, with *r*_s_= 0.629, a *P*-value of < 0.05 in the flexion position and *r*_s_= 0.617, a *P*-value of < 0.05 in the straight position, indicating a significant correlation between the two positions as shown in Figures 4C, D. This suggests that the correlation between the distance of catheter tip displacement and weight gain is higher, especially in the flexion position.

## Discussion

### Premature infant PICC tip displacement

Due to the catheter displacement being highly likely to occur due to natural growth, and the rate of PICC ectopia can be as high as 35% due to factors such as blood flow dynamics and positional changes (20). We found that 100% of premature infants' catheter tips were affected by positional changes in veins with changes in body position and blood flow. After 1 week of placement, we found that the tips of 134 (66.33%)/153 (75.74%) cases are closer to the heart than at placement. This is consistent with Srinivasan's finding that 32.6% of PICC catheters move toward the heart 24 h after placement (21). With the rapid increase in weight and body length, the catheter tip not only did not move away from the heart but also moved closer to the heart. This may be due to catheter bending or folding during placement, followed by flushing with blood flowing toward the heart, resulting in catheter movement toward the heart. Therefore, even after the initial catheter placement is in the ideal position, there is still a high possibility of catheter tip displacement into the heart cavity. Studies have shown that the incidence of PICC-related arrhythmias is 1%, which can lead to the death of children; most arrhythmias occur within the first 2 weeks after insertion of the catheter (22). Therefore, it is essential to use US tracking to determine whether catheter withdrawal is needed 1 week after placement. In addition, for edematous infants, the catheter tip was tracked after the edema subsided, and it was found that the tips of the catheter moved toward the heart. The appearance and disappearance of edema can lead to catheter tip placement that is too shallow or too deep and may enter the atrium. Therefore, US should be used to locate the catheter in a timely manner after edema subsides to determine whether catheter withdrawal is needed. If the tip of the catheter enters the heart, the position would be adjusted. To do this, US was used to measure the length of the catheter tip that has entered into the heart. Then, adjust the tip to the entrance of the atrium, and make a record of the adjustment.

### The relationship between PICC tip displacement and weight gain/length growth in preterm infants

This study showed that the tip of the catheter moved to different degrees with the increase in weight and length and was significantly correlated with weight gain and length growth (*r*_s_ = 0.681/*r*_s_ = 0.629), with a higher correlation with weight gain. Therefore, assessing weight gain is a more accurate predictor of catheter displacement. The ideal position of PICC is the upper and lower thirds of the vena cava. The length of the SVC in preterm infants is only 2–3 cm, while that of IVC is 4–5 cm (18). Therefore, the maximum limit of the ideal position is 1 and 1.67 cm for the SVC and IVC. The results of this study showed that by the third week, the catheter had moved 1.27 ± 0.89 cm, and the catheter of the SVC had moved out of the ideal position. By the fifth week, the catheter had moved 2.23 ± 0.95 cm, and the catheter of the IVC had moved out of the ideal position. At this point, the catheter was considered to have reached the stage where it must be repositioned. If the catheter is left in place and fluid is infused, the inadequate blood flow velocity in the non-central vein can result in insufficient drug dilution, and the high osmotic and high concentration liquid can corrode the venous wall, leading to complications (23, 24). Therefore, catheter displacement should be closely monitored starting from the third and fifth weeks after placement. If timely repositioning is not possible, the safety of the catheter can be confirmed by withdrawing blood, flushing the catheter, and reducing the osmotic pressure of the nutrient solution. Tracking the catheter tip position predictively and removing high-risk catheters as early as possible can maximize the prevention of complications (25, 26), which is also consistent with Costa's view that complications of central venous catheters can be prevented (27).

### The correlation between PICC tip displacement and weight gain/length growth in flexion/straight positions is different

The correlation between PICC tip displacement in flexion/straight positions and weight gain/length growth differed in this study. The correlation was higher in the flexion position; therefore, positioning in the flexion position is recommended. Additionally, from the second week onwards, the tip displacement in the flexion position was 0.23 (0–0.97) cm, while in the straight position, it was 0.25 (0–0.98) cm. Thus, the displacement in the flexion position was smaller, consistent with the correlation results, and positioning in the flexion position is also recommended. Tauzin et al. used US to locate the PICC tip and recommended fixing the position to improve accuracy (7). Therefore, the influence of posture should be fully considered, and the positioning posture should be fixed as the flexion position.

## Limitations

This study has three limitations that need to be addressed in future research. First, the study is based on a limited sample size of superior vena cava catheters from a single center, with no separate analysis of superior and inferior vena cava catheters. A multi-center study with a larger sample size is needed to overcome this limitation, and separate studies should be conducted on the superior and inferior vena cava catheters. Second, there was no large sample study of edematous patients, future research should focus on a dedicated catheter study for patients with edema. Third, a longitudinal study was not carried out to gain an in-depth understanding of the pros and cons of using US in PICC positioning. Future research should address this by conducting a longitudinal study.

## Conclusion

The tip displacement of PICCs in premature infants is significantly correlated with weight gain and length growth. Based on the initial positioning of the catheter, the position should be tracked timely in the first week after placement to determine whether to adjust the catheter position. From the third/fifth week onwards, the frequency of superior/inferior vena cava tracking should be increased. When using US to track the catheter tip, choosing a flexed position is a more reliable method to increase accuracy and consistency.

## Data availability statement

The original contributions presented in the study are included in the article/supplementary material, further inquiries can be directed to the corresponding author.

## Ethics statement

The studies involving human participants were reviewed and approved by the Ethics Committee of Children's Hospital of Chongqing Medical University: No. 2022-369. Written informed consent to participate in this study was provided by the participants' legal guardian/next of kin.

## Author contributions

XT contributed to the acquisition, analysis, interpretation of the data and acquisition, drafting, and final approval of the manuscript. XZ, JW, and YC provided technical support and conceptual advice. XL conceptualized and designed the study, funding acquisition, project administration and supervision, analysis and interpretation of the data, drafted, and critically reviewed the manuscript for important intellectual content. All authors read and approved the final manuscript.
